# The mTOR–lysosome axis at the centre of ageing

**DOI:** 10.1002/2211-5463.13347

**Published:** 2021-12-18

**Authors:** Julian M. Carosi, Célia Fourrier, Julien Bensalem, Timothy J. Sargeant

**Affiliations:** ^1^ Lysosomal Health in Ageing Hopwood Centre for Neurobiology Lifelong Health Theme SAHMRI Adelaide Australia

**Keywords:** ageing, age‐related disease, autophagy, lysophagy, lysosome, mTOR

## Abstract

Age‐related diseases represent some of the largest unmet clinical needs of our time. While treatment of specific disease‐related signs has had some success (for example, the effect of statin drugs on slowing progression of atherosclerosis), slowing biological ageing itself represents a target that could significantly increase health span and reduce the prevalence of multiple age‐related diseases. Mechanistic target of rapamycin complex 1 (mTORC1) is known to control fundamental processes in ageing: inhibiting this signalling complex slows biological ageing, reduces age‐related disease pathology and increases lifespan in model organisms. How mTORC1 inhibition achieves this is still subject to ongoing research. However, one mechanism by which mTORC1 inhibition is thought to slow ageing is by activating the autophagy–lysosome pathway. In this review, we examine the special bidirectional relationship between mTORC1 and the lysosome. In cells, mTORC1 is located on lysosomes. From this advantageous position, it directly controls the autophagy–lysosome pathway. However, the lysosome also controls mTORC1 activity in numerous ways, creating a special two‐way relationship. We then explore specific examples of how inhibition of mTORC1 and activation of the autophagy–lysosome pathway slow the molecular hallmarks of ageing. This body of literature demonstrates that the autophagy–lysosome pathway represents an excellent target for treatments that seek to slow biological ageing and increase health span in humans.

## Graphical abstract

Slowing biological ageing represents a target that could significantly increase health span and reduce the prevalence of multiple age‐related diseases. Inhibition of mTORC1 can slow ageing; however, research on how this happens is still ongoing. We discuss the bidirectional relationship between mTORC1 and its major effector, the autophagy–lysosome pathway and how this relationship can be used to address age‐related disease. Created with BioRender.com.

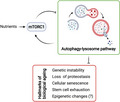

**Abbreviations**
AKT: protein kinase B
AMPK: 5' adenosine monophosphate‐activated protein kinase
ARFGAP1: ADP‐ribosylation factor GTPase‐activating protein 1
ATF4: activating transcription factor 4
ATG: autophagy‐related
BCL2: B‐cell lymphoma 2
BECN1: beclin‐1
CARM1: coactivator‐associated arginine methyltransferase 1
CASTOR: cytosolic arginine sensor for mTORC1
cGAS‐STING: cyclic GMP–AMP synthase–stimulator of interferon genes
CLEAR: coordinated lysosomal expression and regulation
CRAC: cholesterol recognition amino acid consensus
Deptor: DEP domain‐containing mTOR interacting protein
DNAm: DNA methylation
DNASE2: deoxyribonuclease 2
ESCRT: endosomal sorting complex required for transport
FGF21: fibroblast growth factor 21
FIP200: FAK family kinase‐interacting protein of 200 kDa
FLCN: folliculin
FNIP: FLCN‐interacting protein
FOXO3: Forkhead box O‐3
FYCO‐1: FYVE and coiled‐coil domain autophagy adaptor 1
GAP: GTPase‐activating protein
GATOR: GTPase‐activating protein activity towards Rags
GEF: guanine nucleotide exchange factor
GFAP: glial fibrillary acidic protein
GLB1: galactosidase beta 1
hTERT: human telomerase reverse transcriptase
KICSTOR: KPTN, ITFG2, C12orf66, and SZT2 complex
LC3B: microtubule‐associated protein 1A/1B‐light‐chain 3B
LIR: LC3‐interacting region
MAPK: mitogen‐activated protein kinase
MITF: microphthalmia‐associated transcription factor
mLST8: mammalian lethal with SEC13 protein 8
mTOR: mechanistic target of rapamycin
mTORC1: mTOR complex 1
NAD+: nicotinamide adenine dinucleotide
NBR1: neighbour of BRCA1
PI3K: phosphatidylinositol 3‐phosphate kinase
PRAS40: proline‐rich Akt substrate of 40 kDa
Raptor: regulatory‐associated protein of mTOR
RHEB: ras homolog enriched in brain
ROS: reactive oxygen species
SAMTOR: S‐adenosylmethionine sensor upstream of mTORC1
SAR1B: secretion associated related GTPase 1B
SASP: senescence‐associated secretory phenotype
SKP2: S‐phase kinase‐associated protein 2
SLC38A9: solute carrier 38 A9
SQSTM1/p62: sequestosome 1
STAT3: signal transducer and activator of transcription 3
TBC1D7: TBC1 domain‐containing 7
TER: telomerase RNA
TERT: telomerase reverse transcriptase
TFE3: transcription factor E3
TFEB: transcription factor EB
TRIM16: tripartite motif‐containing 16
TSC: tuberous sclerosis complex
UBE2QL1: ubiquitin‐conjugating enzyme E2 Q family‐like 1
ULK1: Unc‐51‐like autophagy activating kinase
VPS34: vacuolar protein sorting 34
WIPI2: WD repeat domain phosphoinositide‐interacting 2

## Age‐related diseases represent a large unmet clinical need

Many countries now have ageing populations, thereby increasing the prevalence of age‐related diseases such as dementia [1]. It may seem obvious, but it is important to acknowledge that ageing is the number one risk factor for age‐related disease. When we talk about ageing in this context, we mean biological ageing, which is the progressive deterioration of physiological function over time. Some people age well and acquire age‐related disease much later in life, whereas some people age poorly and have a shorter health span, the period of life in which an individual is healthy and a shorter lifespan [2, 3]. Whereas traditional approaches in science and medicine have attempted to target the unique pathologies of age‐related diseases in order to slow their progression, it may make more sense to try to slow ageing itself [4]. While it has been known for a long time that nutrient restriction can slow ageing and age‐related disease [5], we are only now beginning to understand the mechanisms through which these interventions work. One of the pathways that is known to mediate at least some of the beneficial effects of nutrient restriction on ageing is through mechanistic target of rapamycin (mTOR) signalling and its interaction with the autophagy–lysosome pathway. mTOR is a serine/threonine kinase that was found to bind a macrolide antibiotic called rapamycin, in the presence of FK506‐binding protein 12 [6]. In this review, we focus on mTOR signalling, its relationship with the autophagy–lysosome pathway, and how this relationship interacts with the fundamental processes that underpin biological ageing.

## The lysosomal system: a network of vesicles for waste recycling

Cells generate ‘waste’ in the form of unwanted or damaged organelles and macromolecules that must be targeted to the lysosome for destruction. In the lysosome, the enzymatic breakdown of carbohydrates, proteins, lipids and nucleic acids is coordinated by various hydrolases which function optimally in this acidic environment. Substrates that are destined for destruction are delivered to the lysosome by endocytosis [7], phagocytosis [8] and autophagy, with the latter being the best characterized in relation to ageing and age‐related disease [9]. As such, we primarily focus on the autophagy–lysosome pathway in this review. Autophagy can be separated into three subtypes: microautophagy, chaperone‐mediated autophagy and macroautophagy. This review focuses on macroautophagy (hereafter referred to as autophagy), which is potently activated by nutrient depletion, cellular stress and infection, but it also occurs to a lesser extent under basal conditions.

Autophagy, through the autophagy–lysosome pathway, is directly inhibited by the nutrient sensing kinase mTOR, within the context of the mTOR complex 1 (mTORC1) [10, 11, 12]. mTORC1 is localized to the lysosome and has very strong interactions with biological ageing. However, mTOR also exists within another complex called mTORC2.

mTORC2 can be found attached to the inner leaflet of the plasma membrane, mitochondria, ribosomes, endoplasmic reticulum and the endolysosomal system [13, 14, 15, 16, 17]. While some data suggest that mTORC2 is regulated by phosphatidylinositol 3‐phosphate kinase (PI3K), its control is complex, nuanced and subject to conflicting data. This topic has been reviewed well elsewhere [15]. In stark contrast to mTORC1, mTORC2 is not acutely sensitive to nutrition or rapamycin [18]. While the majority of this review focuses on mTOR’s impact on the autophagy–lysosome pathway and biological ageing through its association with mTORC1, it is important to note that mTORC2 also plays a role here. mTORC2 is known to suppress autophagy [19] and is also important for healthy ageing [20].

## The lysosomal surface–the home of mTORC1 activation

mTORC1 is a master regulator of cell growth that promotes anabolism in many ways (e.g. 5’ cap‐dependent translation, ribosome biogenesis, nucleotide and lipid synthesis) while also inhibiting catabolism (autophagy). mTORC1 is composed of mTOR in complex with regulatory‐associated protein of mTOR (Raptor), mammalian lethal with SEC13 protein 8 (mLST8), proline‐rich Akt substrate of 40 kDa (PRAS40) and DEP domain‐containing mTOR interacting protein (Deptor) and is recruited to the lysosome when nutrients (such as amino acids, glucose and cholesterol) are abundant.

**Fig. 1:**
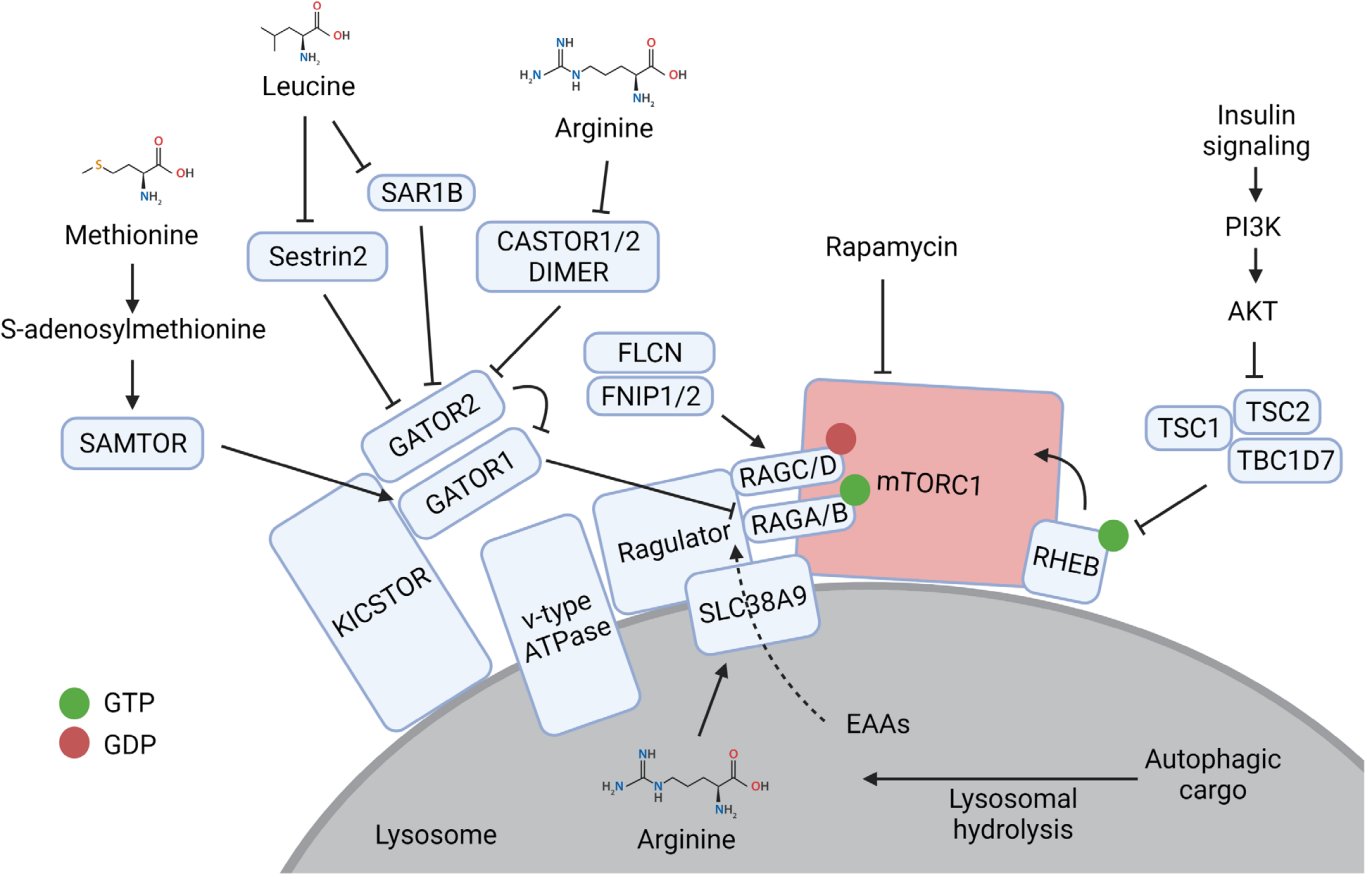
mTORC1 is activated on the surface of the lysosome. Amino acids from the lysosomal lumen and outside of the lysosome signal through known proteins and protein complexes to recruit mTORC1 to the lysosome, where it is activated by insulin signalling through RHEB. Created with BioRender.com.

When amino acids are plentiful, mTORC1 interacts with the RAG GTPases (RAGA or RAGB, and RAGC or RAGD) and together is recruited to the lysosomal surface by a multiprotein complex called Ragulator, which is a guanine nucleotide exchange factor (GEF) that regulates GTP loading of RAGA or RAGB proteins [21, 22] (Fig. 1). In contrast, the folliculin (FLCN) and FLCN‐interacting protein (FNIP) dimer is a GTPase‐activating protein (GAP) for RAGC or RAGD [23]. In order to be recruited to the lysosome, mTORC1 interacts with RAGA^GTP^ or RAGB^GTP^ in complex with RAGC^GDP^ or RAGD^GDP^. The nutrient‐regulated targeting of mTORC1 to the lysosome enables the activation of its kinase activity by the small GTPase ras homolog enriched in brain (RHEB). To regulate mTORC1, amino acids require v‐type ATPase activity, which is a proton pump that acidifies the lysosome and binds to Ragulator, a process that is thought to regulate mTORC1 localisation [24].

The nucleotide status of the RAG proteins is regulated by amino acid availability through a series of multiprotein complexes called GTPase‐activating protein activity towards Rags (GATOR)1, GATOR2 and KICSTOR (KPTN, ITFG2, C12orf66 and SZT2 complex). RAGA and RAGB are maintained in a GDP‐bound state by the GATOR1 complex, which is a GAP that is recruited to the lysosome by KICSTOR [25]. Moreover, GATOR2 binds to and inhibits GATOR1 to enable mTORC1 activation at lysosomes [26].

Amino acids regulate the activity of these protein complexes that orchestrate mTORC1 activity at the lysosome through sensor proteins that are specific to different amino acids and metabolites. Cytoplasmic leucine sensor protein Sestrin2 interacts with and inhibits GATOR2, which in turn prevents GATOR1 recruiting mTORC1 to the lysosome for activation. Leucine binds to Sestrin2 to disrupt the Sestrin2‐GATOR2 interaction and therefore allows GTP loading of RAGA/B and mTORC1 activation [27]. In a similar way, leucine binds to the sensor protein, secretion associated related GTPase 1B (SAR1B), leading to dissociation of the SAR1B‐GATOR2 interaction, and promotes mTORC1 activation [28]. Interestingly, SAR1B and Sestrin2 recognize different parts of the leucine molecule although the affinity of leucine for SAR1B is superior to Sestrin2, indicating that SAR1B is a more potent leucine sensor [28].

The mTORC1 pathway is also stimulated by arginine that is sensed in the cytoplasm by a protein homodimer comprised of cytosolic arginine sensor for mTORC1 (CASTOR)1, or a heterodimer comprised of CASTOR1/2. Analogous to Sestrin2 and SAR1B, homo‐ or hetero‐dimers of CASTOR1/2 inhibit GATOR2 in a manner that is relieved upon binding to arginine [29].

Methionine sufficiency is signalled to mTORC1 through a distinct mechanism via the protein S‐adenosylmethionine sensor upstream of mTORC1 (SAMTOR). Rather than binding to methionine itself, SAMTOR binds to the methionine metabolite S‐adenosylmethionine. SAMTOR interacts with GATOR1 and KICSTOR to potentiate GAP activity against RAGA/B to repress mTORC1 activation in the absence of methionine. In contrast, during methionine sufficiency, S‐adenosylmethionine binds to SAMTOR resulting in its dissociation from GATOR1 and the subsequent activation of mTORC1 [30].

Arginine within lysosomes is sensed by the lysosomal membrane protein and amino acid transporter solute carrier 38 A9 (SLC38A9). Arginine sensing by SLC38A9 promotes its interaction with the Rag‐Ragulator complex on the lysosomal surface to activate mTORC1 [31]. In an arginine‐regulated fashion, SLC38A9 transports many essential amino acids, including leucine, from the lysosomal lumen into the cytoplasm [31]. Independent of its arginine sensing function, but dependent on its cholesterol recognition amino acid consensus (CRAC) motif, SLC38A9 senses cholesterol availability within lysosomes. This activates mTORC1 signalling in a RAG‐dependent manner [32].

In the absence of amino acids, mTORC1 dissociates from the lysosome and is inactive. This process is dependent on ADP‐ribosylation factor GTPase‐activating protein 1 (ARFGAP1). ARFGAP1 binds to mTORC1 to stop lysosomal localisation in the absence of amino acids; overexpression of ARFGAP1 prevents the recruitment of mTORC1 to the lysosomal surface and inhibits downstream signalling, whereas the loss of ARFGAP1 promotes mTORC1 activity, even in the absence of amino acids [33].

Glucose availability is relayed to mTORC1 by at least two different mechanisms: (a) the 5' adenosine monophosphate‐activated protein kinase (AMPK)‐mediated phosphorylation of Raptor and tuberous sclerosis complex 2 (TSC2) in response to glucose deprivation, which inhibits mTORC1 signalling [34, 35, 36] and (b) the glycolysis metabolite dihydroxyacetone phosphate which signals glucose sufficiency to mTORC1 [37]. In the absence of AMPK, glucose deprivation still suppresses mTORC1 signalling, which fails to occur following dihydroxyacetone phosphate supplementation [37]. Collectively, these mechanisms detail how the availability of specific nutrients, both cytoplasmic and within lysosomes, cooperate with a network of regulatory proteins to fine‐tune the lysosomal recruitment of mTORC1 where it is activated by the small GTPase RHEB.

Growth factor cues and nutrient availability must be integrated by mTORC1 to drive a pro‐growth programme. Pro‐growth signals (e.g. insulin and growth factors) are passed onto RHEB via the insulin receptor‐PI3K‐AKT‐TSC1/2/TBC1 domain‐containing 7 (TBC1D7) axis [38, 39, 40] or through mitogen‐activated protein kinase (MAPK) signalling [41]. This means that only in the presence of both nutrients and pro‐growth signals will mTORC1 become activated. It is important to note that the lysosome is the likely site of mTORC1 activation rather than the location from which it signals, which is consistent with the diverse subcellular localisations of mTORC1 substrates [42].

## Lysosomal behaviour modifies mTORC1 signalling

It is noteworthy that the lysosome is not just a platform for mTORC1 signalling, as lysosomal activity and localisation also impact mTORC1 activity. During starvation, lysosomes crowd around the microtubule organizing centre next to the nucleus [43]. During nutrient replete conditions, lysosomes spread to the cell periphery. Manipulation of motor proteins revealed that this localisation changes mTORC1 activity. Peripheral localisation of lysosomes nearer to the plasma membrane increases mTORC1 activity because it is closer to phospho‐active AKT; conversely, perinuclear localisation of lysosomes decreases mTORC1 activity [43]. The peripheral localisation of lysosomes during nutrient replete conditions depends on the production of phosphatidylinositol‐3‐phosphate on the lysosomal surface by vacuolar protein sorting 34 (VPS34) and the binding of this phospholipid by FYVE and coiled‐coil domain autophagy adaptor 1 (FYCO‐1) and protrudin [44]. Further, a recent study found lysosomal positioning impacted not only mTORC1, but also mTORC2 [17]. This is one example of how lysosomal behaviour modifies mTORC1 activity.

Lysosomal activity also impacts mTORC1 signalling. mTORC1 is not only sensitive to signals from the cytosol and plasma membrane, but also to activity in the lysosomal lumen itself. Inhibition of the v‐type ATPase inhibits stimulation of mTORC1 activity by amino acids [24, 45]. The requirement for v‐type ATPase activity lies downstream of amino acids but upstream of RAGA/B; some studies also suggest that mTORC1 also senses amino acids within the lysosomal lumen [24]. Autophagy and the breakdown of protein into amino acids are critical for reactivation of mTORC1 during starvation. Acute nutrient restriction stops mTORC1 from signalling; however, after prolonged restriction, mTORC1 activity is restored by autophagy. In the absence of autophagy‐related gene 7 (ATG7), which is required for ligation of ATG8‐dependent autophagy–see below), mTORC1 is not reactivated following long‐term leucine deprivation [31]. Similarly, mTORC1 activity can also be inhibited by chloroquine [45], which prevents fusion between autophagosomes and lysosomes [46]. Another study reported that lysosomal protease inhibitors E64D and pepstatin could decrease mTORC1 activity [47]. In contrast to these results, reduction of autophagy by beclin‐1 (BECN1–a protein that is critical for the induction of autophagy) knockdown in A549 and HeLa cells does not reduce mTORC1 activity, nor does inhibition of intralysosomal proteolysis by the addition of leupeptin [45]. Although this literature contains some conflicting data, it seems that mTORC1 activity is dependent on lysosomal activity, which is blocked by v‐type ATPase inhibition and some other chemical inhibitors of cargo delivery and proteolysis.

## The passage of macromolecules through the autophagy lysosomal pathway is controlled by mTORC1

**Fig. 2:**
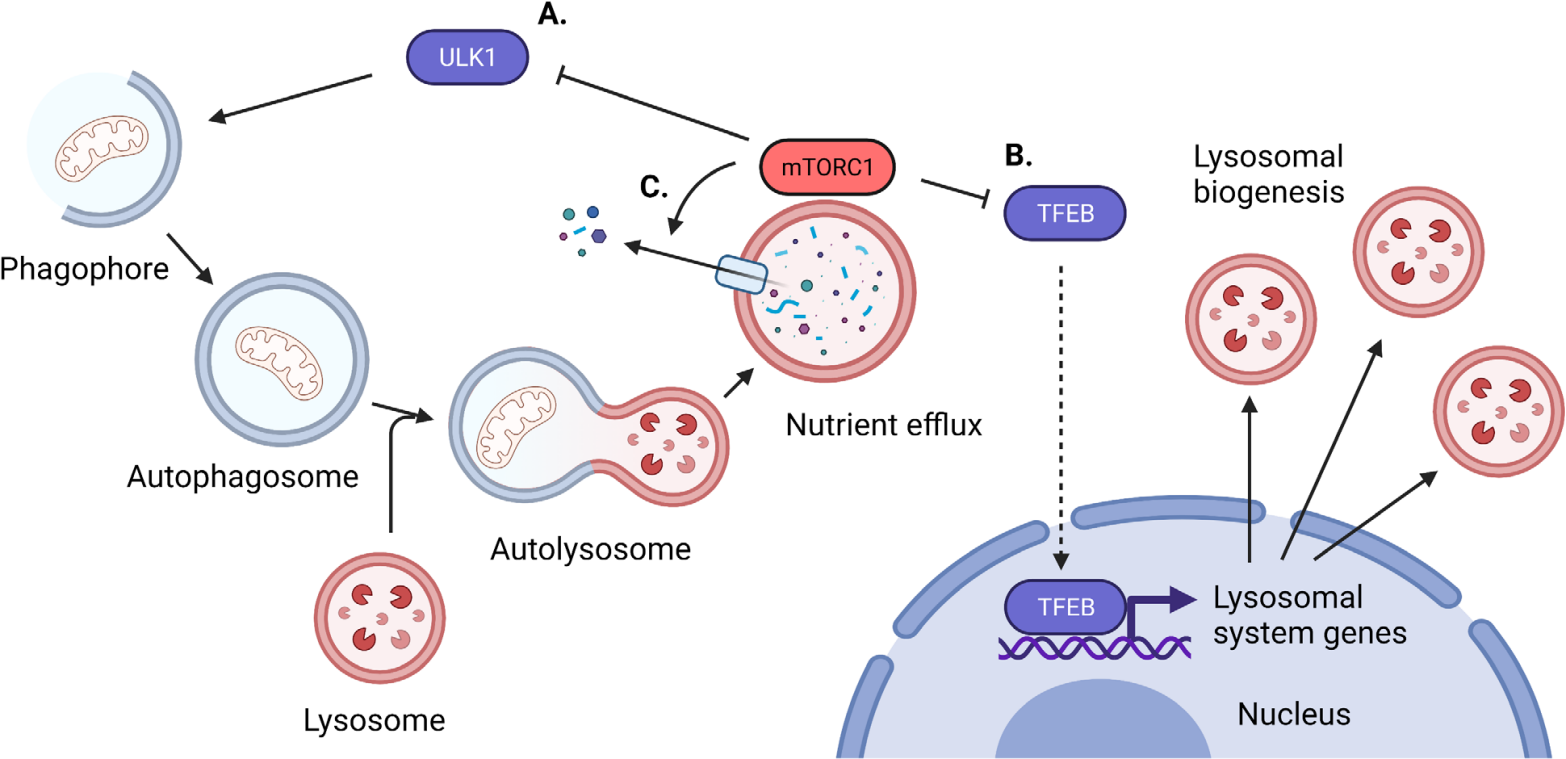
mTORC1 controls multiple aspects of the autophagy–lysosome pathway. (A) mTORC1 inhibits the initiation of autophagy by inhibiting the activity of ULK1. Autophagy in this figure is shown as growth of the phagophore into an autophagosome, its fusion with a lysosome and subsequent nutrient efflux. (B) mTORC1 inhibits lysosomal biogenesis by inhibiting the activity of MITF family transcription factors such as TFEB. (C) Efflux of nutrients generated by the lysosomal hydrolysis of autophagic cargo is dependent on mTORC1 activity. Created with BioRender.com.

mTORC1 activity executes a pro‐growth–and thus, anticatabolic–programme through multiple effectors. One way in which it does this, relevant to the lysosomal system, is to repress initiation of autophagy through phosphorylation of Unc‐51‐like autophagy activating kinase (ULK)1/2 (Fig. 2). ULK1 is a substrate of AMPK and mTORC1 and is required for the initiation of autophagy. It has a paralog called ULK2 with which it shares some autophagy‐related redundancy [48]. However, for the rest of this review, we focus on ULK1. ULK1^S555^ is phosphorylated by AMPK and causes the activation of ULK1 under starvation conditions [49]. Conversely, mTORC1 phosphorylates ULK1^S757^ during nutrient sufficiency which occludes phosphorylation and thus activation by AMPK [50]. Following mTORC1 deactivation, ULK1 initiates autophagy.

Active ULK1 exists in a complex with other proteins (including FAK family kinase‐interacting protein of 200 kDa (FIP200), ATG101, and ATG13 [51, 52]) and initiates autophagy by interacting with downstream protein complexes. Autophagy itself is regulated through known protein complexes. These include ATG9 vesicles, the ATG14‐containing VPS34 complex, the ATG2–ATG18 complex, and the ATG12 and ATG8 conjugation systems [53]. ULK1 is required for the correct localisation of ATG9, which is required for growth of the limiting membrane of the phagophore [54] in conjunction with the ATG2‐ATG18 complex [55, 56]. Growth of the limiting membrane occurs through lipid transfer from the endoplasmic reticulum through ATG2 and recruitment of ATG9‐positive vesicles from the Golgi apparatus. ULK1 also phosphorylates BECN1 to promote activation of the ATG14‐containing VPS34 complex [57], which localizes to the growing phagophore [58]. This generates phosphatidylinositol‐3‐phosphate on the phagophore, which recruits WD repeat domain phosphoinositide‐interacting 2 (WIPI2) and then the ATG12‐ATG5‐ATG16L1 complex to promote the E3‐like transfer of ATG8 proteins (such as microtubule‐associated protein 1A/1B light‐chain 3B [LC3B]) to the lipid phosphatidylethanolamine which decorates autophagic compartments [59]. ATG8 proteins that become lipidated onto the nascent phagophore are required to capture specific cargos via autophagy receptors that contain LC3‐interacting region (LIR) motifs [60]. ATG8 proteins are also required for efficient fusion between the autophagosome and the lysosome [61].

Lysosomal hydrolases degrade macromolecules into their constituent small molecules/monomers such as amino acids, fatty acids and cholesterol. These small molecules are exported back to the cytosol in different ways [62, 63, 64]. Interestingly, efflux of amino acids from the lysosome appears to depend on the proton gradient that is maintained by the v‐type ATPase, mTORC1 activity and the lysosomal arginine sensor SLC38A9. This again demonstrates the very close relationship between mTORC1 and lysosomal activity, all the way from initiation of autophagy to regulating the efflux of catabolites from degradation of autophagic cargos in the lysosome.

## Autophagy–lysosome pathway gene expression is regulated by mTORC1

Lysosomal system genes are regulated by numerous transcription factors including but not limited to signal transducer and activator of transcription 3 (STAT3) [65, 66]. Activating transcription factor 4 (ATF4) [67] and microphthalmia‐associated transcription factor (MITF)‐family transcription factors (including transcription factor (TF)E3, TFEB and MITF) [68, 69, 70]. mTORC1 works through the MITF family transcription factors to control lysosomal system gene expression [69, 71, 72]. Under nutrient replete conditions, MITF family transcription factors are recruited to the lysosome where they are phosphorylated by mTORC1 [69, 70]. This causes binding of a 14‐3‐3 protein to these transcription factors and inhibits their translocation to the nucleus. When nutrients are lacking, mTORC1 activity is reduced, leading to TFEB activation and nuclear entry. This also requires calcium efflux from the lysosome to enable calcineurin‐dependent dephosphorylation of TFEB [73]. TFEB phosphorylation by mTORC1 is sensitive to amino acid withdrawal but insensitive to growth factor withdrawal, making it an unusual mTORC1 substrate (other substrates require the coincidence of both nutrient signalling through the RAGs and insulin signalling through RHEB on mTORC1 to be phosphorylated by this complex) [74]. Once in the nucleus, MITF family transcription factors bind to coordinated lysosomal expression and regulation (CLEAR) elements in the promoters of autophagy and lysosomal system genes to increase their expression. This ultimately augments lysosome biogenesis and catabolism [75]. To prepare for the regeneration of free amino acids generated via catabolism during starvation, TFEB stimulates the expression of RAGD to prime the mTORC1 pathway for reactivation [76].

## Age‐related lysosomal damage–a direct emergency signal between the lysosome and mTORC1

Although the lysosome provides a platform for mTORC1 signalling in response to nutrition, nutrition‐independent signalling also impacts mTORC1 signalling on the lysosomal surface. Age‐related phenomena, such as lysosomal membrane permeabilization, can also have a large impact on mTORC1 signalling. Another direct route of communication between lysosomes and mTORC1 exists to prevent defective lysosome accumulation.

Age‐related diseases can lead to the build‐up of molecules that can puncture the lysosomal membrane, which is dangerous for the cell. Lysosomal membrane damage can result in leakage of lysosomal proteases that cause cell death in both physiological [66, 77] and pathological situations [78]. Such age‐related diseases include atherosclerosis (through accumulation of cholesterol crystals) [79], gout (through uric acid crystals) [80] and late‐onset neurodegenerative diseases (through fibrils of amyloid proteins comprised of tau, amyloid‐β and α‐synuclein, for example) (reviewed in [81]).

The hallmarks of Alzheimer’s disease include extracellular amyloid plaques and intraneuronal tau tangles, both of which comprise amyloid fibrils that are capable of puncturing the lysosome [82]. Tau, for example, nucleates and grows via a prion‐like mechanism [83, 84]. Seeding of tau aggregates requires acidified endolysosomes [85], from which corrupted tau aggregates escape and spread into the cytosol to propagate more tau aggregates. Not only can the escape of prion‐like amyloids from the lysosome promote their spread, but lysosomal membrane permeabilization also promotes activation of the inflammasome and inflammation [81]. However, the damaged lysosome can also activate a danger signal that initiates its repair or its own removal (termed lysophagy).

Lysosomal membrane damage from harmful substances may initially be repaired. Calcium release from the lysosome, and a β‐galactoside‐binding lectin called galectin 3, triggers recruitment of endosomal sorting complex required for transport (ESCRT) proteins to damaged lysosomal membranes [86, 87]. ESCRT proteins are better known for their role in sorting proteins in the endolysosomal system; however, they also participate in membrane repair [88]. Recruitment of ESCRT proteins to the lysosome is necessary for repair of lysosomal integrity and re‐acidification [87].

If damage to the lysosomal membrane cannot be repaired, the lysosome must be removed via lysophagy. This is possible through mTORC1. Damage to lysosomal membranes is a powerful inducer of autophagy and inhibits mTORC1 signalling to an extent similar to nutrient starvation [89]. Several studies have identified the molecular mechanisms that underpin the communication between damaged lysosomes and mTORC1. Galectin 8 is critical for mTORC1 inactivation by lysosomal membrane damage. During normal conditions, galectin 8 is in proximity to mTOR. However, upon lysosomal membrane damage, galectin 8 shifts to be in proximity to SLC38A9 and Ragulator components. At the same time, mTORC1 leaves the lysosomal membrane and becomes inactive. Importantly, galectin 8 is required for silencing mTORC1 activity in response to lysosomal membrane damage but not for reducing mTORC1 activity during starvation [90]. The same study demonstrated that another galectin family member, galectin 9, is critical for activation of AMPK in response to lysosomal damage. Galectin 3 recruits autophagic machinery around the terminally damaged lysosome. This process, which ends in lysophagy of the damaged lysosome, involves recruitment of the E2 ubiquitin‐conjugating enzyme, UBE2QL1 (ubiquitin‐conjugating enzyme E2 Q family‐like 1) [91], E3 ubiquitin ligase TRIM16 (tripartite motif‐containing 16) [92], ubiquitination of lysosomal membrane proteins [93] and recruitment of the segregase P97 [94]. Galectin 8‐mediated mTORC1 inhibition promotes TFEB‐driven expression of lysosomal system genes to replace the lysosomes that were destroyed [86].

This system, which senses and responds to lysosomal system damage, inspired Jia and colleagues [90] to speculate that surveillance of endomembrane damage through the galectins could explain why mTORC1 localizes to the lysosome. The lysosomal localisation of mTORC1 acts as a defence against lysosomal damage from pathogenic molecular species such as aggregated tau [85] as well as invading microbes such as *Mycobacterium tuberculosis* that also translocate into the cytosol from the lysosomal compartment [95]. The same argument can be made about mTORC1 activation, its need for nutrient sensing and amino acids that originate from lysosomal hydrolysis.

**Fig. 3:**
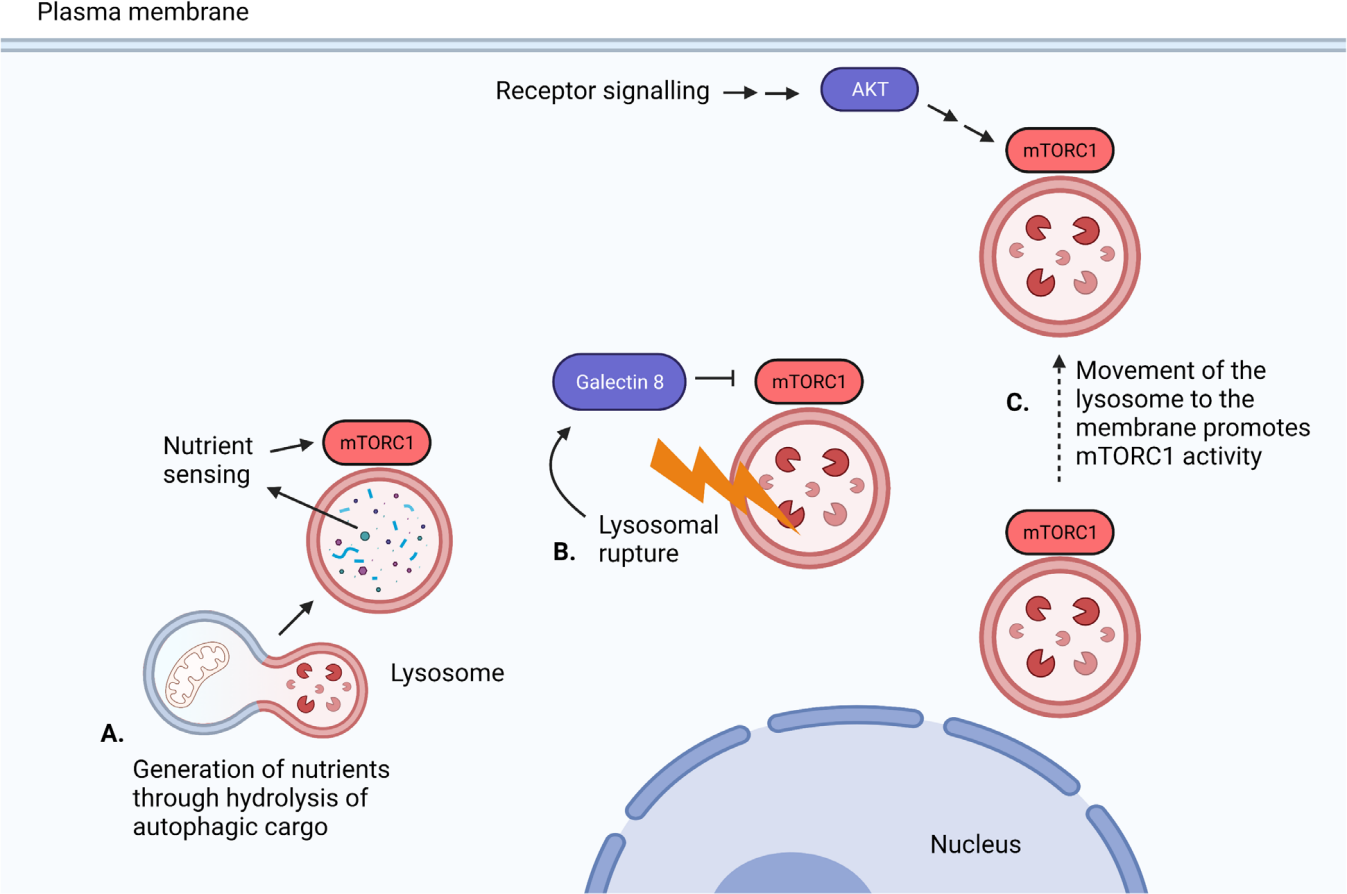
Lysosomal behaviour modifies mTORC1 activity. (A) Generation of nutrients inside the lysosome from hydrolysis of autophagic cargo promotes mTORC1 activity through nutrient sensing. (B) Rupture of the lysosomal membrane suppresses mTORC1 activity through Galectin 8. (C) Movement of the lysosome to the periphery of the cell promotes mTORC1 activity through active AKT signalling. Created with BioRender.com.

Collectively, these studies show that mTORC1 is highly sensitive to lysosomal damage. This provides another example of how lysosomal behaviour influences mTORC1 activity (Fig. 3). mTORC1 inhibition can then orchestrate an appropriate response to lysosomal membrane damage induced by age‐related diseases in the form of crystals and amyloids. The role of mTORC1 inhibition on the autophagy–lysosome pathway and maintenance of the lysosomal system in the face of damage from age‐related pathologies is entirely consistent with its role in ageing in animal models.

## Living longer and healthier through inhibition of mTORC1

Inhibition of mTORC1 activity has long been known to increase both health‐ and lifespan and to slow biological ageing [96]. Experiments from the 1930s showed that rats exposed to calorie restriction lived for longer compared with rats that consumed an *ad libitum* diet [97]. This effect has widely been replicated in diverse organisms from yeast and worms through to mammals [98]. More recently, two studies assessed the impact of calorie restriction on lifespan in Rhesus monkeys: while one study showed beneficial effects of calorie restriction on lifespan [99, 100] and the other did not [101], in aggregate they appeared to support the idea that calorie restriction could produce health benefits in primates [102]. More recently, increases in health‐ and lifespan have also been observed in both humans and animal models with reduction of protein intake to within healthy ranges [103, 104].

Both calorie restriction and protein restriction are known to inhibit mTORC1 signalling [105, 106]. However, other pathways, including AMPK, forkhead box O‐3 (FOXO3), sirtuin, nicotinamide adenine dinucleotide (NAD+) and fibroblast growth factor 21 (FGF21)‐signalling, also have important effects downstream of nutrient restriction [107]. Further, the direct role that mTORC1 inhibition plays in mediating the effects of caloric restriction is subject to conflicting data [107]. Research has shown that target of rapamycin (TOR) inhibition was not additive with calorie restriction in yeast [108] and *C. elegans* [109], but it was in drosophila [110]. What this means is that TOR inhibition was revealed to be an effector of calorie restriction in yeast and *C. elegans* but not in the study that used drosophila. That said, pharmacological mTORC1 inhibition with rapamycin is sufficient to slow biological ageing as it extends lifespan in a wide range of organisms, including mammals [111]. In summary, although mTORC1 inhibition may not account for all longevity benefits of calorie restriction, it is sufficient to promote longevity itself.

Inhibition of mTORC1 is thought to extend lifespan by increasing translational accuracy [112, 113] and by augmenting autophagy and lysosomal biogenesis. The notion that the autophagy–lysosome pathway is a direct effector of TOR inhibition for the extension of lifespan was shown in model organisms including drosophila [110] and *C. elegans* [114].

Further evidence for a role of the autophagic–lysosomal pathway in controlling lifespan in mice comes from experiments in which autophagy was genetically disabled. Whereas body‐wide deletion of core autophagy machinery genes (e.g. *ATG5*) results in death within a few months from generalized tissue degeneration, other experiments have employed more subtle approaches. Heterozygous deletion of *Becn1* reduces lifespan and increases tumour prevalence [115]. Inducible depletion of ATG5, even for a period of only four months, resulted in the increased prevalence of tumours. Conversely, a *Becn1* knock‐in mutation (F121A), which reduced BECN1’s association with anti‐apoptotic protein B‐cell lymphoma 2 (BCL2) and constitutively increased autophagy throughout the lifetime of the mouse, increased health and lifespan. Further, inhibition of autophagy exacerbates disease‐specific signs in a wide range of age‐related disease models. This topic has been reviewed extensively elsewhere [9, 116]. Although age‐related diseases such as Alzheimer’s disease, atherosclerosis and cancer manifest in different ways, common age‐related mechanisms can be found between them that are known as the hallmarks of ageing [117]. In the following section, we review specific examples of how the autophagy–lysosome pathway could lead to a longer life through attacking molecular drivers of ageing. Here, we review specific examples of how mTOR and the autophagy–lysosome system interact with genetic instability, telomere attrition, epigenetic alterations, loss of proteostasis, cellular senescence and stem cell exhaustion [117].

## Genetic instability is suppressed by the autophagy–lysosome system

mTORC1 inhibition and the autophagy–lysosome pathway has long been known to modify diseases that are characterized by genetic instability [118], including cancer [119]. The autophagy–lysosome pathway has a complex relationship with cancer: decreased autophagy before onset of cancer is known to result in increased accumulation of DNA damage and increases the risk of cancer development [115, 120]. However, once a cancer is established, this relationship becomes significantly more complex [121, 122] and has been reviewed elsewhere [123].

The autophagy–lysosome pathway supports genetic stability at different levels. One of these is suppressing the generation of reactive oxygen species (ROS) that would otherwise damage DNA [124]. This occurs by mitophagy, the selective uptake of damaged mitochondria. For example, knockdown of ATG5 using a doxycycline‐inducible shRNA‐expressing mouse (which produces a decrease in ATG8‐dependent autophagy) increased the abundance of mitochondrial content and the incidence of telomere‐associated γ‐H2AX foci, which represent DNA damage that associates with ageing [120]. A similar relationship has been shown using genetic manipulation of multiple core autophagy genes [120, 125, 126], although ROS may not be the only mechanism through which autophagy deficiency promotes the accumulation of DNA damage [127].

The autophagy–lysosome pathway also opposes DNA damage by capturing and digesting nuclear material via nucleophagy [128]. The lysosome degrades nuclear material with a range of hydrolases including deoxyribonuclease 2 (DNASE2). DNASE2 deficiency promotes inflammation and autoimmunity via the cyclic GMP–AMP synthase (cGAS)–stimulator of interferon genes (STING) pathway [129]. The cGAS‐STING pathway is activated by foreign DNA in the cytoplasm (e.g. during bacterial infection) and coordinates the gene expression of innate immune system effectors; it also activates autophagy (i.e. to counteract pathogen invasion). The loss of DNASE2 promotes autoimmunity and inflammation because nuclear DNA is not efficiently cleared by the autophagy–lysosome axis and accumulates in the cytoplasm. Together, these data demonstrate multiple ways that the autophagy–lysosome pathway counteracts DNA damage.

## Telomere attrition has a complex relationship with mTORC1

Telomeres are repetitive DNA sequences that exist at the ends of eukaryotic chromosomes. At each somatic cell division cycle, telomeres shorten through incomplete synthesis of the lagging strand during DNA replication. This is due to the inability of DNA polymerase to completely replicate the 3′ end of the DNA strand [130]. However, telomeres can be stabilized by telomerase, a reverse transcriptase enzyme responsible for adding DNA to the ends of chromosomes, thus maintaining chromosome length. Telomerase is a specialized reverse transcriptase RNP composed of two main components, a telomerase reverse transcriptase (TERT) protein and a noncoding RNA component (TER, telomerase RNA), which is an integral and essential part of the enzyme [131]. Attrition of telomere length has been associated with DNA damage response, which can lead to cellular senescence or apoptosis and genome instability. Further, decreased telomere length has been shown to associate with increased mortality in large human cohort studies [132].

Telomere maintenance is biologically linked to autophagy through TERT. TERT increases expression of autophagy protein LC3 and may increase autophagy in an mTORC1‐dependent manner [133, 134, 135, 136]. However, how augmentation of the autophagy–lysosome pathway interacts with telomere length awaits further research. Although the effect of modulation of autophagy and the lysosomal system on telomere length has not been well explored, the links between nutrient restriction, mTORC1 and telomeres have.

The relationship between mTORC1 and telomeres has been examined in cell models where rapamycin shortens telomeres by decreasing human TERT (hTERT) expression [137, 138]. In a more recent mouse study, mTORC1 inhibition in animals genetically modified to have shorter telomeres actually decreased lifespan. The same study showed that while rapamycin likely increased autophagy in control mice, the same increase was not observed in mice with short telomeres [139]. Further, rapamycin treatment that extended lifespan in wild‐type mice did not change telomere length in the same animals. This research shows that autophagy is unlikely to impact telomere length.

## Epigenetic changes have a bidirectional relationship with mTORC1 and the autophagy–lysosome pathway

Epigenetic control of gene expression can occur through DNA methylation and histone methylation and acetylation, both of which are known to regulate expression of autophagic and lysosomal genes [140]. For example, an E3 ubiquitin ligase S‐phase kinase‐associated protein 2 (SKP2) ubiquitinates the methyltransferase coactivator‐associated arginine methyltransferase 1 (CARM1) to earmark it for degradation in the proteasome (a protein complex that degrades proteins) under nutrient replete conditions. However, during starvation, AMPK activates FOXO3 via phosphorylation, which downregulates SKP2 expression and promotes CARM1‐dependent histone dimethylation (H3R17Me2) to promote the expression of autophagy and lysosomal genes in conjunction with TFEB [141]. While the epigenetic regulation of autophagy is well understood, the interaction between autophagy and age‐related epigenetic changes awaits exploration. However, we can learn from research that has examined the relationship between epigenetic modification and mTORC1.

Apart from histone methylation, DNA itself can be methylated at cytosine bases to form 5‐methylcytosine. DNA methylation can change with various cell activities such as silencing retroviral elements, regulation of gene expression, genomic imprinting, cellular proliferation and X chromosome inactivation [142, 143]. During ageing, patterns of DNA methylation change in a predictable way. Methylation at a specific subset of CpG islands can be used to predict chronological age at a very high accuracy, much more so than for telomere shortening [144].

Interestingly, and contrary to intuition, DNA methylation age (DNAm age) does not correlate well with cellular proliferation, cellular senescence or DNA damage [145]. Despite this, rapamycin does slow biological ageing, measured as the DNAm age [145]. The ability of rapamycin to slow DNAm age could work through autophagy. However, experiments that test this idea have not yet been conducted and this remains a gap in the literature.

## The autophagy–lysosome pathway is critical for suppressing accumulation of damaged proteins with age

During ageing, the autophagy–lysosome pathway is critical for maintaining proteostasis in tissues. Proteostasis includes protein synthesis, which is directly regulated by mTORC1 [112], and protein degradation, which is performed by the proteasome and the lysosomal system. The autophagy–lysosome pathway is particularly important for clearance of insoluble and aggregated proteins that cannot be cleared by the proteasome [146]. The role of the autophagy–lysosome pathway in the maintenance of proteostasis is most evident in late‐onset neurodegenerative diseases which are often referred to as proteinopathies. As an organism ages, the autophagy–lysosome pathway is thought to become less efficient, although this has not been directly tested in humans [147, 148]. This reduction in cellular clearance results in the accumulation of misfolded and aggregated proteins, with some of these entering an ‘amyloid state’. This is true for Alzheimer’s and Parkinson’s diseases where amyloid‐β peptide [149], tau [150] and α‐synuclein [151] accumulate and spread in a strain‐specific [151] prion‐like manner. In Alzheimer’s disease, accumulation of amyloid‐β starts 20 years before the diagnosis of dementia [152]. Although late‐onset neurodegenerative diseases are complex and multifactorial, protein aggregation and prion‐like spread are thought to drive at least some decline in cognition and function with disease progression.

mTORC1 inhibition with drugs such as rapamycin represses this accumulation and the prion‐like spread of toxic proteins [153]. The autophagy–lysosome pathway is critical for suppression of these corrupted protein species. Whereas tau monomer is a long‐lived protein, with a half‐life of over six days *in vitro* and three weeks in the human central nervous system [154], tau aggregates respond acutely to autophagic inhibition [154]. Lysosomal inhibition with chloroquine promotes the accumulation of tau aggregates *in vivo*. The process whereby protein aggregates are selectively captured by autophagy is referred to as aggrephagy [155]. Aggrephagy may occur through ubiquitin‐dependent and ubiquitin‐independent recognition of protein aggregates by autophagy‐cargo receptors such as sequestosome 1 (SQSTM1)/p62 and neighbour of BRCA1 (NBR1). Autophagy cargo receptors tether the aggregate to the growing phagophore via binding to ATG8 family proteins and conclude with the destruction of aggregates in the lysosome [156]. Therefore, the autophagy–lysosome pathway suppresses protein aggregation and is likely to be a key vulnerability factor in proteinopathies such as Alzheimer’s disease.

As mentioned above, it is thought that the activity of the autophagy–lysosome pathway deteriorates with age. Research on protein ageing has provided a possible mechanism for deterioration of protein degradative capability within the lysosome. Nonenzymatic modifications of amino acids can yield peptides that cannot be cleaved by lysosomal proteases. These modifications include the deamidation of L‐asparagine or the isomerisation of L‐aspartic acid. Through known intermediates, this process proceeds spontaneously under physiological conditions and results in the generation of modified amino acids such as D‐aspartic acid, D‐isoaspartic acid and L‐isoaspartic acid [157]. Importantly, the amyloid‐β peptide is susceptible to this kind of modification on its own aspartic acid residues [158], making these modified peptides resistant to lysosomal proteases [159]. Such resistance to proteolysis will promote the accumulation of these peptides in the lysosome and may explain why the lysosomal system is compromised in brains containing amyloid plaques [160, 161]. Spontaneous amino acid isomerisation within peptides is therefore a phenomenon that contributes to the deterioration of the role of the lysosome in proteostasis during ageing.

## Cellular senescence is suppressed by the autophagy–lysosome pathway

During ageing, tissues accumulate senescent cells that contribute to inflammation and tissue degeneration. Cellular senescence is characterized by withdrawal from the cell cycle, dysregulated metabolism, macromolecular damage and a defined secretory phenotype (called the senescence‐associated secretory phenotype, or SASP) [162]. mTORC1 inhibition suppresses cellular senescence. Rapamycin reduces cellular senescence, despite not being senolytic–instead it allows cells to re‐enter the cell cycle [163, 164, 165]. Further, autophagy suppresses the accumulation of senescent cells in mouse tissues [120]. However, the relationship between autophagy and age‐related senescence is complex because the autophagy–lysosome pathway supports the synthesis of the SASP, which also requires mTORC1. Therefore, the autophagy–lysosome pathway itself may be responsible for generation of the SASP through the provision of amino acids for synthesis of this senescence hallmark [166, 167].

Senescent cells also depend on the lysosomal system in other ways. They display increased lysosomal mass and this accounts for an increase in β‐galactosidase (expressed by the lysosomal gene, galactosidase beta 1 (*GLB1*)), which is often used as a marker of senescent cells [168, 169]. Further, total histone content decreases in senescent cells which extrude chromatin that is cleared by autophagy [170]. This process may be linked to tumour suppression. Together, these studies show that inhibition of mTORC1 and activation of the autophagy–lysosome pathway may be clinically useful for the repression of senescence before large numbers of cells have already become senescent.

## Stem cell exhaustion is repressed by autophagy

Another important hallmark of ageing is stem cell exhaustion. During ageing, stem cells lose their ability to respond to tissue damage, lose control over proliferative activity and experience functional decline. This loss of function reduces their ability to support tissue repair and replacement [171].

A relationship between autophagy and stem cell maintenance has recently emerged whereby an active autophagy–lysosome pathway maintains their ability to proliferate. This relationship has been characterized for neuronal stem cells, in which autophagy appears to decrease during ageing [172]. Autophagy in neuronal stem cells is driven, at least in part, by the transcription factor FOXO3 [173]. Loss of FOXO3 in these cells promotes the accumulation of protein aggregates, consistent with reduced autophagy. Rapamycin can reverse this phenotype, consistent with the role of mTORC1 in suppressing autophagy. Similarly, autophagy inhibition in glial fibrillary acidic protein (GFAP)‐expressing neuronal progenitors via FIP200 deletion also results in accumulation of autophagic proteins, higher cellular content of mitochondria that appear more heterogeneous than in controls and increased amounts of reactive oxygen species [174]. Mice that are deficient for autophagy in neuronal stem cells also had fewer neurons in the dentate gyrus of the hippocampus, which relies on neurogenesis throughout adult life from nearby stem cells. This effect was rescued using antioxidant treatment, showing oxidative damage was likely causing this deficiency in neurogenesis [174].

In support of data showing that a decrease in autophagy reduces neuronal stem cell proliferation, autophagy–lysosome pathway activation can increase the ability of stem cells to proliferate. This is true of quiescent neuronal stem cells that are exposed to nutrient deprivation, rapamycin or overexpression of TFEB [175]. Genetically increasing autophagy via *Becn1* F121A knock‐in also increases the number of neuronal stem cells in the brain in old, but not young mice [172], indicating that autophagy delays stem cell exhaustion [172]. This is consistent with hematopoietic stem cells where increased autophagy in old age maintains mitochondrial health and long‐term regeneration potential [176]. These studies show that efficient autophagy–lysosome pathway function is critical to slow stem cell exhaustion and promote efficient tissue regeneration into old age.

## Interventions that increase activity of the autophagy–lysosome pathway

Activation of the autophagy–lysosome axis is possible, at least in cell and animal models. It is very important to make a clear distinction here between two potential therapeutic strategies–induction of autophagy and enhancing lysosomal activity. Induction of autophagy here refers to an intervention that increases autophagy initiation, sequestration of autophagic cargo and its delivery to the lysosome. Inducers of autophagy have been reviewed elsewhere [177] but include a wide variety of mTOR‐ and mTORC1‐inhibiting drugs such as rapamycin and AZD2014 [178]. In addition to these pharmaceutical compounds, some natural products can also modify mTOR activity such as curcumin and resveratrol [179, 180]. Other drugs, such as lithium, metformin, spermidine and a peptide that interacts directly with autophagy machinery–TAT‐BECN1 [177, 181], can also promote autophagy via mTORC1‐independent mechanisms. Similarly, as mentioned previously in this review, caloric or protein restriction has also been shown to inhibit mTOR activity.

Enhancing lysosomal activity refers to interventions that enhance the acidification and/or the hydrolytic capability of the lysosome. This distinction is important because during age‐related diseases such as Alzheimer’s disease, lysosomal activity is significantly impaired [161] and inducing autophagy in this situation could cause more harm than good [153]. The reason for this is because increasing the generation of autophagic cargo in the absence of efficient lysosomal disposal can cause autophagic stress, which is known to cause cell dysfunction and death. Reports of lysosomal activity inducers are fewer in number than for autophagy inducers; however, some have been developed.

The small molecule EN6 that binds to the v‐type ATPase promotes lysosomal acidification while interfering with Rag‐dependent mTORC1 recruitment and activation at the lysosome. Therefore, EN6 is unique in its ability to simultaneously activate autophagy while directly boosting lysosomal function [182]. mTORC1 inhibition will also enhance lysosomal system function indirectly, via TFEB/MITF‐mediated expression of important lysosomal genes such as subunits of the v‐type ATPase. The MITF family of transcription factors are known to increase lysosomal function, although other transcription factors can perform analogous tasks. STAT3 is an important transcription factor that can dramatically increase lysosomal function through expression of lysosomal genes [65, 66]. Importantly, STAT3‐dependent induction of lysosomal gene expression may be possible via inhibition of a lysosomal protease called asparagine endopeptidase (or legumain) [65]. Another interesting avenue for research into restoration of lysosomal function is delivery of acidic nanoparticles, which appear to rescue compromised lysosomal function [183, 184]. There is clearly much promise for autophagy–lysosome pathway intervention, and while the mTORC1 and related pathways look like good candidates for development of therapies, autophagy–lysosome‐modifying strategies clearly extend well beyond mTORC1.

## Conclusions and perspectives

Autophagy‐based interventions, including via mTORC1 inhibition, hold great promise for slowing biological ageing and delaying age‐related disease. Much has been learned about the basic mechanisms that drive autophagy, its effects on the hallmarks of ageing, and how it can be targeted using both pharmacological and dietary interventions. Even though the lysosome–autophagy pathway is now at a place where it is attracting attention from many research laboratories and even the private sector [185, 186], translation of this knowledge to promote healthy ageing has not yet been realized.

Research must now focus clearly on questions that will allow translation of interventions that enhance the activity of the autophagy–lysosome pathway. A particular emphasis should be put towards measuring the activity of the autophagy–lysosome pathway in humans. Our knowledge about how this pathway operates in healthy human ageing and age‐related disease is very limited and this is holding translation back [187, 188]. Although little is currently known, research has now demonstrated that measurement of the activity of the autophagy–lysosome pathway is possible in humans [189].

Another vital question that we need to answer before interventions targeting the autophagy–lysosome pathway can be applied to people is at which stage(s) in human health and disease will augmenting the activity of this pathway be helpful for slowing the accumulation of the hallmarks of ageing? One study provided great insight with regard to proteostasis. In mouse models of Alzheimer’s disease, rapamycin treatment applied before the accumulation of tau protein tangles prevented their accumulation. However, when applied after the emergence of tau tangles, rapamycin had no effect [190]. Examination of the relationship between the autophagy–lysosome pathway and other hallmarks of ageing raises similar questions. This pathway appears to suppress the emergence of senescent cells in tissues [120] but is also required to support key elements of the senescent phenotype [166, 167]. The autophagy–lysosome pathway appears important for suppressing genetic damage but once autophagy is suppressed and this genetic damage appears, it is irreversible [120]. This leads us to speculate that augmenting the activity of the autophagy–lysosome pathway may only be useful before the onset of age‐related diseases, not after. However, the answer to whether this is true will only ultimately come from further research on the autophagy–lysosome pathway in humans.

## Conflict of interest

TJS and JB are listed as inventors on a related patent, PCT/AU2020/050908.

## Author contributions

TJS conceived and wrote the manuscript. JMC, CF, and JB drafted and edited the manuscript.
